# Influence of coracoclavicular heterotopic ossifications in treatment of clavicular fractures: Case report

**DOI:** 10.1007/s00113-026-01686-y

**Published:** 2026-02-12

**Authors:** Malik Jessen, Philipp Zehnder, Peter Biberthaler, Chlodwig Kirchhoff, Markus Schwarz

**Affiliations:** 1 Department of Trauma Surgery, Klinikum rechts der Isar, TUM University Clinic, Technical University Munich, Ismaninger Str. 22, 81675 Munich, Germany; 2 Department of Sports Orthopedics, Klinikum rechts der Isar, TUM University Clinic, Technical University Munich, Ismaninger Str. 22, 81675 Munich, Germany

**Keywords:** Coracoclavicular ligaments, Trauma, Biomechanical phenomena, Stability, Lig. coracoclaviculare, Trauma, Biomechanische Phänomene, Stabilität

## Abstract

**Background:**

Heterotopic ossification (HO) arising from inflammatory processes often results in bony transformation of soft tissue. While the occurrence of HO is well-documented, its biomechanical influence on clavicular fractures remains less explored. We present a case report of a patient with a clavicular fracture following a bicycle accident, accompanied by incidental findings of HO around the coracoclavicular (CC) ligaments.

**Case presentation:**

A 59-year-old man presented with a displaced right-sided clavicular fracture and a cervical vertebral body fracture following a bicycle fall. Incidental findings revealed pronounced HO around the CC ligaments. The patient underwent surgical stabilization for the cervical vertebral body fracture and clavicular fracture using open reduction and internal fixation. The presence of ossified CC ligaments may influence the biomechanical considerations for clavicular fractures.

**Conclusion:**

The present case report suggests that ossified CC ligaments may impact clavicular biomechanics, potentially offering increased stability at the CC attachment and acting as a protective shield during trauma. This observation may indicate a possible predisposition to fractures in the clavicular region medial to the CC ligaments. While we cannot determine causality based on this single case, pronounced ossification of the CC ligaments could increase local stiffness and alter load distribution.

## Abstract (de)

**Hintergrund:**

Heterotope Ossifikation (HO) aufgrund von Entzündungsprozessen führt häufig zu einer knöchernen Umwandlung von Weichgewebe. Während das Auftreten von HO in verschiedenen Szenarien gut dokumentiert ist, ist ihr biomechanischer Einfluss auf Klavikulafrakturen noch wenig erforscht. Wir präsentieren den Fallbericht eines Patienten mit einer Klavikulafraktur nach einem Fahrradunfall, begleitet von zufälligen Befunden einer HO im Bereich der korakoklavikularen (CC-)Bänder.

**Falldarstellung:**

Ein 59-jähriger Mann stellte sich mit einer verschobenen Fraktur der rechten Klavikula und einer Fraktur des Halswirbelkörpers nach einem Sturz mit dem Fahrrad vor. Zufällige Befunde zeigten eine ausgeprägte HO um die CC-Bänder herum. Bei dem Patienten wurde eine chirurgische Stabilisierung der Halswirbelkörperfraktur und der Klavikulafraktur mittels offener Reposition und interner Fixation durchgeführt. Das Vorhandensein ossifizierter CC-Bänder kann die biomechanischen Überlegungen bei Klavikulafrakturen beeinflussen.

**Schlussfolgerung:**

Der vorliegende Fallbericht legt nahe, dass ossifizierte CC-Bänder die Biomechanik des Schlüsselbeins beeinflussen können, möglicherweise eine erhöhte Stabilität an der CC-Befestigung bieten und bei Traumata als Schutzschild fungieren. Diese Beobachtung könnte auf eine mögliche Prädisposition für Frakturen im Schlüsselbeinbereich medial der CC-Bänder hinweisen. Obwohl wir anhand dieses einzelnen Falls keine Kausalität feststellen können, könnte eine ausgeprägte Ossifikation der CC-Bänder die lokale Steifigkeit erhöhen und die Lastverteilung verändern.

## Background

In general, inflammatory processes lead to bony transformation of soft tissue, considered as heterotopic ossification (HO) [1]. HO can occur after direct local muscular trauma, surgical procedures like arthroplasty, spinal cord and head injuries, and mechanical ventilation [2, 3]. A history of trauma is present in approximately 75% of the cases; for the remaining 25% an unrecognized trauma is suggested [4]. If HO does not present with any clinical symptoms, no further treatment is necessary.

On the other hand, clavicular fractures often result from falls, such as those caused by bicycle accidents [5]. In most cases, clavicle fractures occur as midshaft injuries. Lateral clavicle fractures account for approximately 10–30% of all clavicle fractures and are frequently associated with disruption of coracoclavicular (CC) ligaments [6–8].

Despite presuming that HO alters the biomechanical properties of the CC ligaments, the potential influence on a clavicular fracture has not yet been analyzed in detail. Therefore, we present our biomechanical considerations by describing a case report of a patient suffering from a clavicular fracture following a bicycle accident, along with incidental findings of HO around the CC ligaments.

## Case presentation

**Fig. 1:**
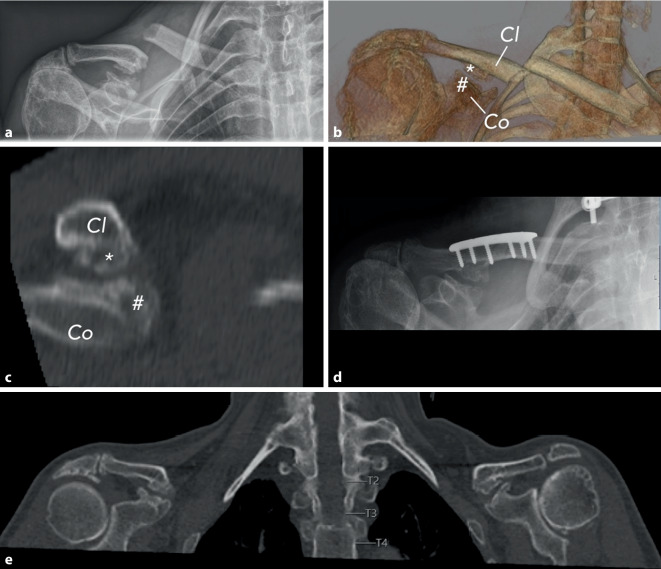
**a** Preoperative conventional anteroposterior X‑ray of the right shoulder demonstrating the displaced clavicular fracture. **b** Three-dimensional computed tomography (CT) reconstruction of the right shoulder illustrating the fracture morphology (clavicle [Cl], coracoid process [Co], ossified portions of the coracoclavicular ligaments apically [*], and caudally [#]). **c** Sagittal CT reconstruction illustrating the spatial relationship between the clavicle, coracoid process, and the ossified coracoclavicular ligaments. **d** Postoperative anteroposterior X‑ray following open reduction and internal fixation with plate osteosynthesis. **e** Coronal CT image of both shoulders showing that the contralateral shoulder demonstrates comparable heterotopic ossification of the coracoclavicular ligaments

**Fig. 2:**
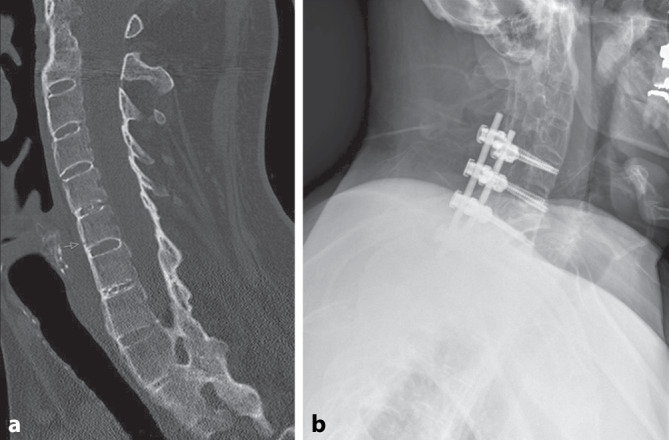
**a** Preoperative sagittal computed tomography (CT) image of the cervical spine demonstrating a Bechterew-associated B2 fracture of the C6 vertebral body according to the AO Spine classification. **b** Postoperative lateral radiograph showing dorsal instrumentation from C5 to Th1 following CT-navigated screw placement, confirming correct alignment and stable fixation

A 59-year-old, right-dominant-handed, Caucasian man had a simple bicycle fall on his back and right shoulder. The initial diagnostics in our university emergency department revealed a displaced right-sided midshaft clavicular fracture (Fig. 1, B1.3 according to OTA/AO classification) and a fracture of the 6th cervical vertebral body (Fig. 2, B2 according to AO spine classification). Medical history of the patient included ankylosing spondylitis, Crohn’s disease, and a lumbar vertebral body replacement due to spondylodiscitis.

X‑ray and CT imaging yielded additional incidental findings in terms of pronounced HO in the cranial and caudal aspects of the CC ligaments. The patient reported no previous pain, although his right shoulder was limited in range of motion (120° flexion and abduction, 20° external rotation, internal rotation up to the 12th thoracic vertebra following the hand-behind-back method) due to a nontraumatic, degenerative supraspinatus tendon lesion a few years ago. No previous accidents involving the right shoulder were reported, and the patient was not restricted in either professional or leisure activities due to his right shoulder problems.

The fracture of the cervical vertebral body was treated by surgical stabilization (Fig. 2). Six days after the trauma, the clavicular fracture was treated with open reduction and internal fixation using locking plate osteosynthesis (Fig. 1). Due to the preoperative asymptomatic condition of the HO and the intraoperatively evaluated stability of the CC ligaments, the HO was neither resected nor stabilized. A clinical evaluation of the patient 6 months postoperatively revealed the same pain-free shoulder function as before surgery.

## Fracture description and biomechanical considerations

The clavicular fracture was located medially to the clavicular attachment of the CC ligaments in terms of a transverse fracture (Fig. 1). Thus, it represents a fracture in the transitional zone between a lateral and shaft fracture, classified as B1.3 according to the OTA/AO classification or type IIA according to the classification by Neer.

The ossification of the CC ligaments measured 6.3 mm at the clavicular apical attachment and 5.9 mm at the coracoidal caudal attachment, as determined by CT (Fig. 1). The gap between the two ossification sites measured 1.8 mm, indicating a free interval from the HO. Therefore, the CC ligaments appeared to be partially ossified. CT revealed incidental HO of the CC ligaments with a similar extent on the opposite side (Fig. 1). Measurements were as follows: 6.0 mm apical attachment, 6.1 mm caudal attachment, 2.5 mm gap between the two attachments.

**Fig. 3:**
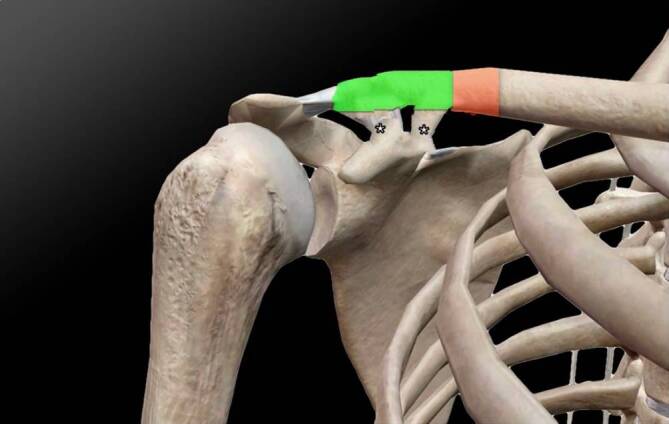
Outline of the right shoulder, adapted from [17]. The green zone represents the area of increased stability of the clavicle, while the red zone represents the area of decreased stability. The asterisks (*) mark the ossified coracoclavicular (CC) ligaments

Direct force applied to the clavicle, such as in the case of a bicycle accident, often leads to rupture of the CC ligaments [5]. Due to the presence of ossifications at both the apical and caudal CC ligament attachments (Fig. 1), a stabilizing effect of both CC ligament attachments can be presumed. The structural importance of the CC ligaments in transmitting load between the clavicle and scapula has been demonstrated in several biomechanical studies, which report substantial stiffness, viscoelastic behavior, and high failure loads of the native CC ligament complex [9–13]. These data support the theoretical assumption that structural alterations such as ossification may influence local force distribution. Therefore, a fracture in the region of the CC ligament attachments appears unlikely (Fig. 3, green zone) if the clavicle exhibits a higher stabilizing effect at the CC ligament attachments compared to native CC ligaments without HO. However, in the presence of ossified CC ligaments, the clavicular region medial to the CC ligament attachments may represent a potential fracture site (Fig. 3, red zone). This consideration remains hypothetical and cannot be derived from this single case alone; a dedicated biomechanical evaluation would be required.

## Discussion

Our case report suggests that ossified CC ligaments have a biomechanical influence on the clavicle. Thus, HO formation at the CC ligaments could provide more stability at the clavicular CC attachment, with the result of a possible protective shielding of the CC ligaments themselves during trauma to the clavicle (Fig. 3, green zone).

In the case of trauma to the clavicle, the load is not transmitted through the CC ligamentous part, but to the biomechanically more unstable part of the clavicle, located directly medial to the CC attachment (Fig. 3, red zone).

A fracture in the region of the CC ligaments with rupture of the ligaments seems unlikely in the setting of ossification of the ligaments.

HO in the region of the CC ligaments has previously been described as a rare incidental finding without clinical symptoms [14–16]. In 1970, Villány made the first description of ossification of the CC ligaments, describing an asymptomatic case without the need for treatment [16].

In 1990, Chen et al. described a retrospective case series of 36 patients with 46 calcifications around the CC ligaments as incidental findings on chest X‑rays [14]. The ossifications ranged from a punctate appearance to larger, periarticular ossifications around the CC ligaments. A review of the clinical history revealed the occurrence of HO on CC ligaments, especially after previous trauma to the clavicle or renal failure.

Qureshi et al. reported ossification of CC ligaments in a paraplegic patient [15]. Interestingly, in contrast to previous publications and our presented case report, the CC ligaments were completely ossified without any intermittent gaps.

To the best of our knowledge, the presented case report represents the first description of a clavicle fracture in the presence of ossified CC ligaments.

To verify our hypothesis that ossified CC ligaments exhibit increased biomechanical stability following direct trauma to the clavicle and subsequent fracture, a biomechanical study would be required. Thus, only presumptions can be made based on the presented case report.

## Conclusion

The present case report suggests that ossified CC ligaments may impact clavicular biomechanics, potentially offering increased stability at the CC attachment and acting as a protective shield during trauma. This observation may indicate a possible predisposition to fractures in the clavicular region medial to the CC ligaments. While we cannot determine causality based on this single case, pronounced ossification of the CC ligaments could increase local stiffness and alter load distribution.

## Abbreviations

CC: Coracoclavicular
CT: Computed tomography
HO: Heterotopic ossification
OTA/AO: Orthopaedic Trauma Association/Arbeitsgemeinschaft für Osteosynthesefragen

## Declarations

For this article no studies with human participants or animals were performed by any of the authors. All studies mentioned were in accordance with the ethical standards indicated in each case. For images or other information within the manuscript which identify patients, consent was obtained from them and/or their legal guardians.
